# Lethal and sublethal heat-exposure of bed bugs (*Cimex lectularius* L.) causes alarm pheromone emission and elicits a movement response in nearby recipients

**DOI:** 10.1038/s41598-024-57925-y

**Published:** 2024-04-12

**Authors:** Aaron R. Ashbrook, Jeffrey L. Feder, Gary W. Bennett, Matthew D. Ginzel, Ameya D. Gondhalekar

**Affiliations:** 1 Department of Entomology, Louisiana State University, 404 Life Sciences Building, Baton Rouge, LA 70803 USA; 2 Department of Biological Sciences, 100 Galvin Life Science Center, University of Notre Dame, Notre Dame, IN 46556 USA; 3 Department of Entomology, Purdue University, 901 West State Street, West Lafayette, IN 47901 USA

**Keywords:** Animal behaviour, Entomology

## Abstract

Many gregarious insect species use aggregation and alarm pheromones. The bed bug, *Cimex lectularius* L., emits an alarm pheromone (AP), a 70/30 blend of (*E*)-2-hexenal and (*E*)-2-octenal, when threatened. Bed bugs avoid temperatures above 43 °C, which are lethal to bugs and used commercially as spatial heat treatments to manage infestations. However, the interaction of bed bug AP in heat avoidance has not been investigated. The goal of this research was to: 1) determine if bed bugs emit AP as an alarm response to heat exposure, and 2) quantify the behavioral responses of conspecifics to AP emitted by heat-exposed bed bugs. Using a selected ion flow tube mass spectrometer, we found that bed bugs responded to lethal and sublethal heat exposure by emitting AP. The Harlan laboratory population emitted more pheromone than a laboratory adapted field population from Florida (McCall). Harlan females emitted the most AP, followed by Harlan males, McCall females and males. In separate behavioral experiments, we showed that conspecifics (i.e., recipients) reacted to AP released by heat exposed bed bugs (i.e., emitters) by frantically moving within 50 mm and 100 mm test arenas. The Harlan recipients reacted to AP in 100 mm areas, whereas the McCall strain did not, indicating a short area of effectiveness of the AP. Synthetic AP components tested in behavioral experiments caused identical effects as the natural AP blend released by heat-exposed bed bugs.

## Introduction

Insects use semiochemicals for communicating intra- or interspecifically^1,2^. Allelochemicals elicit a response in other species, whereas pheromones act between conspecifics^1^. Pheromonal communication can be more important than visual, tactile, or auditory modes of communication^3^. Volatile pheromones can be specific blends of chemical compounds, which are active at low concentrations and capable of traveling long distances^3^. Sex pheromones are often produced by females and are used for insect management either for attracting pests to traps or population monitoring^4,5^. When pheromones are used by insects to communicate danger to conspecifics they are termed ‘alarm pheromones’ (abbreviated as AP)^6^.

Alarm pheromones are often produced in greater quantities than sex pheromones and can serve a variety of functions, including defense induction, activity inhibition, aggregation, repellency, or agitation behavior^7–9^. When threatened, social insects utilize AP, which causes a rapid increase in nestmate activity in the area of release^2,9–11^. The concentration of AP can influence the responses of conspecifics or recipients (insects that are responding to emitted pheromones). At low concentrations, *n*-undecane and formic acid cause the black carpenter ant, *Camponotus pennsylvanicus* D., to aggregate*,* but at high concentrations, formic acid acts as an AP^12^. Similarly, workers of the neotropical stingless bees, *Trigona subterranea* F., are attracted to low concentrations of citral, but high concentrations act as an AP^8^.

Hemipteran insects also emit AP when threatened. For example, hemipterans from the families Pentatomidae, Alydidae, Rhopalidae, and Coreidae emit different combinations of either (E)-2-hexenal, (E)-2-octenal, 4-oxo-(E)-2-hexenal, or 4-oxo-(E)-2-octenal in response to threats such as predation^13–17^. The commonality and similar function of these aldehyde blends (e.g., hexenal and octenal) within Hemiptera indicates their evolutionary conservation in certain families^16,17^. The kissing bug, *Triatoma infestans*, emits an AP composed primarily of isobutyric acid with 2-butanone and 2-methylbutyric acid as minor components^18^. In response to the threat of predation, at least 10 different species of aphids emit (E)-β-farnesene, which causes nearby conspecifics to stop feeding and leave the area^19^. The emission of AP by aphids to cause dispersal of conspecifics has been suggested to be favored by kin selection^8,19^, whereby emitters (defined as insects that release pheromones to communicate a message) are signaling to genetically-related individuals to disperse from aggregations when in danger.

Bed bugs (Hemiptera: Cimicidae: *Cimex lectularius* L.) are similar to aphids in that they are highly related to each other within infestations^20^ and adults emit an AP as a 70/30 blend of (E)-2-hexenal and (E)-2-octenal (referred to as hexenal and octenal) in response to a threat^21–23^. Bed bug nymphs emit a slightly different blend of AP components, with hexenal and octenal being the main components and 4-oxo-(E)-2-hexenal and 4-oxo-(E)-2-octenal being minor components in an approximate 20/60/15/5 ratio^24,25^. Bed bugs are repelled by the AP blend, moving quickly away from the area of emission in search of safe locations^22,23^. Alternatively, at low concentrations, the bed bug aggregation pheromone blend (hexenal, octenal, and dimethyl disulfide, dimethyl trisulfide, and (E)-2-hexanone) with the addition of histamine^26–29^, has an arrestant effect over short distances on the movement of conspecifics.

The documented situations where bed bugs have been shown to emit AP are: (i) when they are suddenly uncovered, (ii) physically crushed, (iii) exposed to high concentrations of CO_2,_ (iv) contacted with abrasive dust or (v) being chewed by a bat^21,22,30–32^. Adult bed bugs in glass petri dishes have been shown to be repelled from filter paper discs treated with synthetic hexenal and octenal^30^. Adult male bed bugs and 4th or 5th instar nymphs will emit AP in response to being mounted by an adult male to avoid damage from traumatic insemination^33^, but females do not^34^.

Bed bugs are also repelled by other stimuli, such as high temperatures^35–44^ and, if they manage to successfully escape lethally heated areas, pest management outcomes can be negatively impacted^38–44^. Hypothetically, if exposure to high temperature also stimulates live bed bugs to emit AP, then conspecifics that detect the pheromone signal may attempt to flee from a heated area to safety. As a result, the flight behavior in response to AP may contribute to bed bugs surviving heat treatments^42-44^. Alternatively, it is possible that bed bugs would disperse from their harborage in response to AP and get exposed to lethal air temperatures (> 50 °C) during heat treatments as harborages are insulated from higher temperatures during the heating process^42^. In this regard, the authors and other pest management professionals have reported smelling bed bug AP during heat treatments, which led to this study. However, this observation has not been empirically tested and thus it is unknown if bed bugs emit AP in response to heat exposure. The goal of the current laboratory-based study was to test if AP facilitates the flight response of bed bugs to detrimental temperatures. To accomplish this goal, we measured the concentrations of hexenal and octenal emitted by adult male and female bed bugs while being exposed to lethal and sub-lethal temperatures using a selected ion flow tube mass spectrometer (SIFT-MS). We then conducted behavioral bioassays to determine how live bed bugs responded to AP released from nearby heat killed conspecifics. We report that bed bugs emit AP in response to lethal/sublethal heat exposure and nearby conspecifics detect the AP and flee.

## Results

### Emission of bed bug AP after exposure to lethal heat

**Figure 1:**
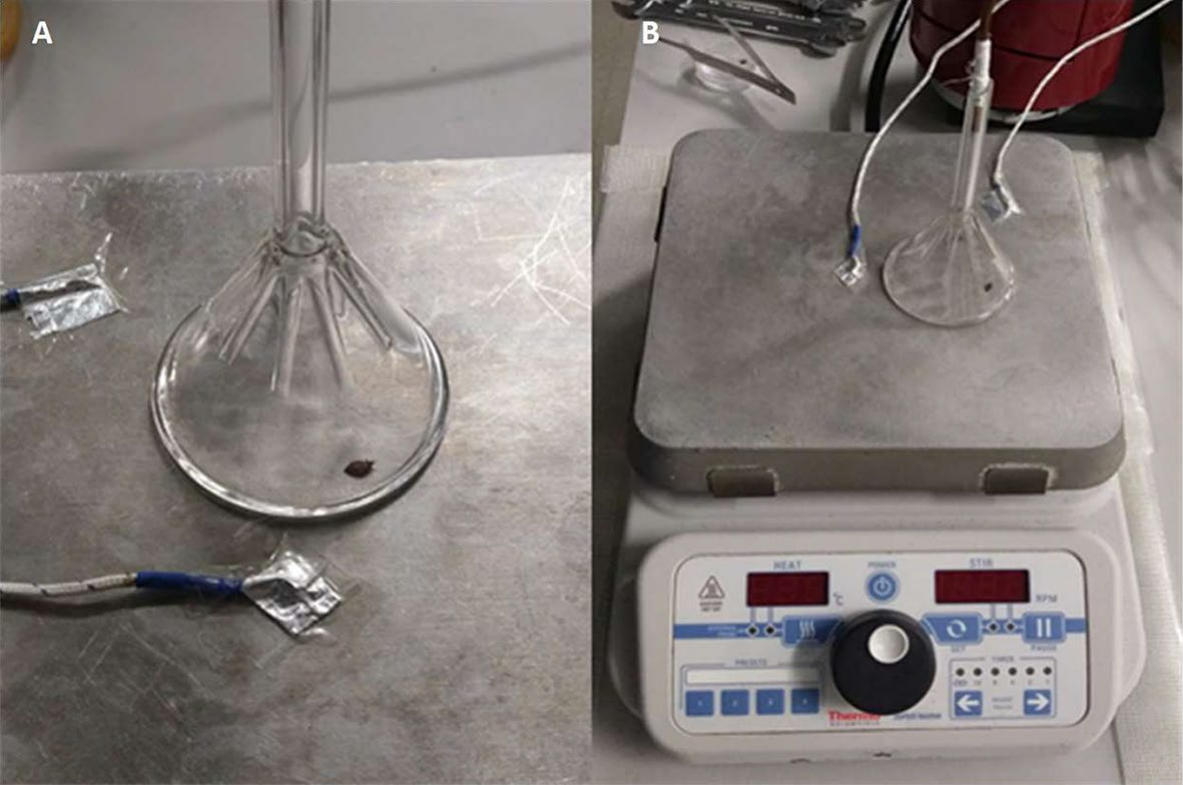
Experimental apparatus used for bed bug volatile pheromone detection. (**A**) Two thermocouples are attached on the left and right of a glass funnel, allowing for temperature recordings of the hotplate. (**B**) Air samples are taken in by the SIFT-MS through the stem of the Pyrex funnel. One adult bed bug is shown placed under an inverted 50 mm Pyrex funnel for alarm pheromone collection.

**Figure 2:**
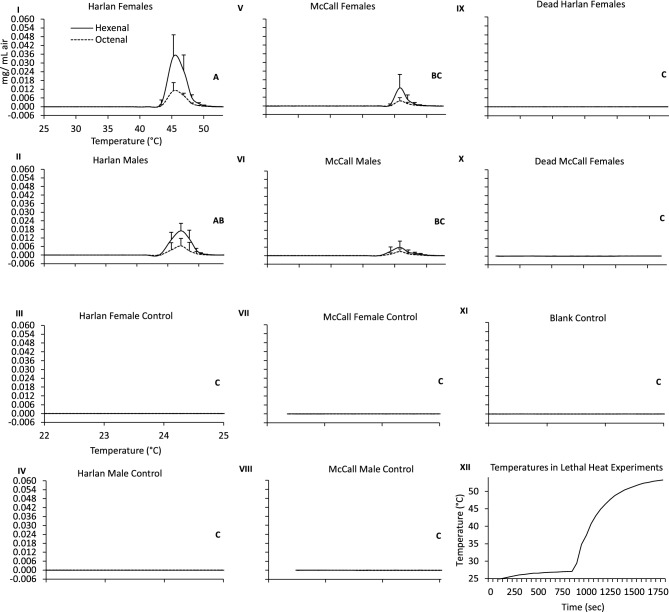
Alarm pheromone (AP) emission from living heated (I) Harlan females; (II) Harlan males; and lack of AP detected from living unheated (III) Harlan female controls; (IV) Harlan male controls. (V) McCall females; (VI) McCall males represent AP from heated living insects, whereas (VII) McCall female controls; (VIII) McCall male controls are from living unheated bed bugs. Dead heated (IX) McCall female (X) Harlan female bed bugs and (XI) empty blank control. Solid lines represent hexenal and dotted lines represent octenal (mg/mL air). Temperatures that insects were heated to in the glass funnels during the lethal heat exposure experiments over time are shown in panel XII. Treatments that do not share the same letters have statistically different concentrations of AP (ANOVA, Tukey’s test, P < 0.05). Error bars represent standard deviation values.

A series of experiments using SIFT-MS were conducted to determine if bed bugs exposed to ramp up heating (25–55 °C in 15 min) in a hot plate—glass funnel apparatus (Fig. 1) emit AP. To determine the extent to which laboratory adaptation might impact pheromone concentrations, bed bugs from two populations were used in the experiment to test for AP emission in response to high temperature exposure. The Harlan bed bug population originally collected from New Jersey has been reared in the laboratory since 1973 and was used as a baseline for making comparisons. The McCall bed bug population fromFlorida was collected from the field in 2016 and, thus, may more closely resemble natural populations despite being reared in the laboratory for ~ 20 generations before use. For both the Harlan and McCall populations, lethal heat exposure induced bed bugs to emit AP (Fig. 2). During the temperature ramp-up phase from room (25 °C) to a lethal temperature (55 °C), both living females and males emitted significantly higher levels of hexenal and octenal compared to all control types prior to heat-induced death (ANOVA: aldehydes*treatment group, df = 11, F-ratio = 8.8, P < 0.0001). Dead bed bugs frozen 24 h before experimentation and returned to room temperature before testing functioned as a biologically relevant control and had similar levels of AP as the control blank glass funnel (Fig. 2). In addition, AP signal did not increase for unheated living bed bugs held in glass funnels as room temperature or 25°C for 15 min (Fig. 2), which served as a second biologically relevant control, ruling out non-heat related stress as the cause for AP release.

Analysis of headspace volatile collections from individual bed bugs revealed that heat exposure consistently resulted in AP emission at temperatures between 44 and 45 °C (Fig. 2). There was no statistical difference in the temperature that caused AP to be emitted by bed bugs of different treatment groups by sex or population (ANOVA: df = 1, F-ratio = 2.7, P = 0.09; SI Table 1 for full ANOVA results). Males in the Harlan population began to release AP at a mean ± STD, temperature of 45.8 ± 0.3 °C and stopped at 51.7 ± 0.2 °C, whereas males from the McCall population emitted AP at 45.1 ± 0.4 °C and stopped at 51.3 ± 0.2 °C (Fig. 2). Harlan females released AP at a mean temperature of 44.7 ± 0.4 °C and ended at 50.1 ± 0.1 °C, whereas McCall females released AP at 44.6 ± 0.5 °C and ended at 51.2 ± 0.1 °C (Fig. 2). Bed bugs emitting AP in the lethal heat experiments appeared motionless and had their legs curled in a manner suggesting heat induced death was impending or had occurred. Twenty-four hours after exposure to the lethal temperature of 55 °C, all bed bugs used in the experiment were dead (mortality was 100%).

Despite showing a similar temperature for emitting AP, there were significant differences in the quantities of AP released by the two populations, with the Harlan population emitting more AP than the McCall population (ANOVA, P < 0.0001; Fig. 2). There were sex related differences in the quantity of AP emitted; Harlan female bed bugs emitted the most AP, with peak measurements of emitted hexenal and octenal were 0.035 ± 0.015 and 0.01 ± 0.005 mg/ mL of air, respectively. Harlan males, McCall females and then McCall males followed in the quantities of AP emitted.

### Emission of bed bug AP after exposure to sublethal heat

**Figure 3:**
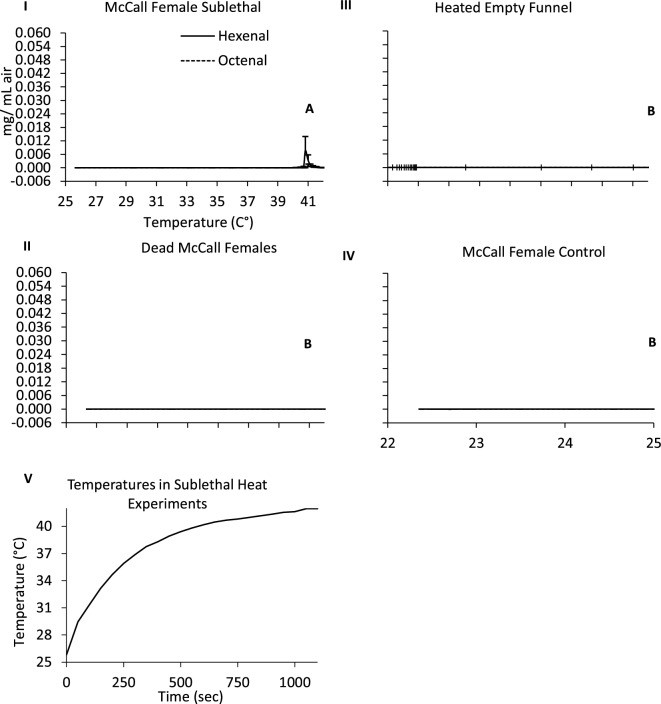
Alarm pheromone (AP) emission of living female McCall female bed bugs (I) in response to sublethal heat exposure. No AP was detected in heated (II) dead McCall females, (III) in empty blank funnel controls, or (Iv) unheated living McCall females. Blue lines represent hexenal and orange lines represent octenal (mg/mL air). (V) Temperatures insects in glass funnel experienced over time during the sublethal heat experiments. Graphs not sharing the same letters have statistically different quantities of hexenal and octenal (ANOVA, Tukey’s test, P < 0.05). Error bars represent standard deviation values.

McCall females were also subjected to ramp-up temperatures from 25 to 42 °C to evaluate whether bed bugs release AP after exposure to sublethal heat at 42 °C. Controls used for these sublethal exposure experiments were identical to those used for lethal heat exposure tests. McCall females in the sublethal heat experiments began to emit AP in a pulse beginning at 40.2 ± 0.1 °C that lasted for ~ 300 s before pheromone concentrations returned to and remained at baseline levels until the experiment was terminated after 8 min (Fig. 3). When the bed bugs emitted AP in the sublethal heat experiment, they were observed to be lying dorsally and erratically moving their legs, suggesting that sublethal heat exposure had led to knockdown. Most bed bugs survived sublethal heating and were alive 24 h (5/6 = 83.3%) after exposure, suggesting that AP is actively released from living bugs in response to sufficient heat stress. There was a significant difference in the quantities of AP detected between the sublethally heated living McCall females, and the biologically relevant and blank controls, i.e., unheated living McCall females, frozen dead bed bugs that were subsequently heat-exposed, and the empty hot plate-glass funnel treatment (ANOVA, P < 0.05 for all treatments). Post-hoc Tukey’s tests detected significant differences in AP quantities emitted from sublethally heated bed bugs versus all three of the control treatments (Fig. 3).

### Behavioral responses of individual and multiple recipients to AP emitted by heat-exposed emitter bed bugs in 50 mm funnels

A series of behavioral experiments on bed bugs at rest were conducted in a Petri dish-glass funnel experimental apparatus (SI Fig. 1) to determine the extent to which live bed bugs (single or in groups of 5) reacted to AP emitted from flame heated conspecifics. Recipient bed bug reaction was quantified as “excited as well as repelled” or “not excited and not repelled” by the emitter or control individual, as described in Levinson & Bar^30^. The reaction of recipient bed bugs to AP from heat killed bed bugs can be visualized in SI video 1. In these studies, recipient bed bugs in the funnel were observed and scored for their movement in a binary manner (1 = yes, if they moved or reacted, 0 = no if they did not move or react).

For the 1:1 emitter to recipient ratio experiments, significantly more insects responded in the treated (living bed bugs exposed to AP emitted by flame heated bugs) than control group (living bed bugs exposed to previously frozen dead bugs) for both populations (Logistic regression, P < 0.05, Table 1A). Individual recipients responded to emitter bed bugs by moving rapidly. These experiments confirmed that bed bugs at rest will react to the AP only from bed bugs that were alive prior to being heated.

**Table 1:** Percentages of replicates where bed bugs (recipients) did or did not respond by moving in response to AP in behavioral assays, during the 5 min observation period.

	Yes, responded (%)	No, did not respond (%)	N	DF	Chi-square	P-value
(A) Heated living bugs, 1 emitter: 1 recipient, 50 mm						
Control Harlan males	5	95	40	1	17.9	< 0.0001
Living heated Harlan males	65	35				
Control Harlan females	5	95	40	1	30.2	< 0.0001
Living heated Harlan females	85	15				
Control McCall males	0	100	40	1	33.8	< 0.0001
Living heated McCall males	80	20				
Control McCall females	5	95	40	1	15.8	0.0002
Living heated McCall females	60	40				
(B) Heated living bugs, 1 emitter: 5 recipients						
Control Harlan males	47	53	42	1	7.9	0.0063
Living heated Harlan males	90	10				
Control Harlan females	10	90	42	1	21.5	< 0.0001
Living heated Harlan females	86	14				
Control McCall males	43	57	42	1	23.6	< 0.0001
Living heated McCall males	100	0				
Control McCall females	5	95	42	1	30.2	< 0.0001
Living heated McCall females	86	14				
(C) Heated living bugs, 1 emitter: 1 recipient, 100 mm						
Control Harlan males	11	89	40	1	4.8	0.032
Living heated Harlan males	44	56				
Control Harlan females	5	95	38	1	4.86	0.031
Living heated Harlan females	39	61				
Control McCall males	22	78	36	1	0.81	0.36
Living heated McCall males	11	88				
Control McCall females	5	95	34	1	0.36	0.54
Living heated McCall females	22	78				

Controls are compared to living heated bed bugs using logistic regression (P < 0.05). Part A of the table shows results from the 1:1 ratio emitter: recipient assays performed in 50 mm glass funnels. Part B shows 1:5 ratio emitter: recipient results in 50 mm glass funnels. Part C shows the 1:1 ratio emitter: recipient results in 100 mm glass funnels. In all experiments, the recipients were the same sex and from the same population as the emitter.

The 1:5 emitter to recipient study also displayed a significant difference in the number of responding recipient bed bugs between the control and treated groups (Logistic Regression, P < 0.05; Table 1B). Emitter bed bugs elicited identical movement in recipients as described in the abovementioned experiments, but in multiple responding conspecifics.

### Testing active range of AP in 100 mm funnels

After determining that bed bugs will respond to AP emitted from living flame heated insects, we investigated the active space or range of effectiveness of the AP signal by repeating the 1:1 emitter: recipient experiments in a larger 100 mm glass funnel (8.4 × greater volume than the 50 mL funnel). A significant difference was observed between treated and control groups for the Harlan population (P < 0.05), but not for the McCall population (P > 0.05) (Table 1C). The percentages of Harlan and McCall recipients that reacted to AP emitted by heated bed bugs was overall lower in the experiments with a 100 mm funnel (Logistic regression, response of heated emitters in small funnels compared to response in large funnels, N = 163, DF = 1, Chi-square: 30.23, P < 0.0001).

### Emission of AP and its characterization from living and dead bed bugs

To determine the extent to which high temperature exposure from flame heating caused emission of AP in both living and dead bed bugs prior to use in behavioral assays, separate SIFT-MS experiments were conducted. As expected, significant differences in the quantities of emitted AP were detected between living and dead flame heated bed bugs, with essentially no AP detected from dead insects (SI Fig. 2, ANOVA, Tukey’s test, P < 0.001). As seen in the lethal AP detection experiments, differences were also found between Harlan and McCall populations and between males vs. females for living flame heated bed bugs (SI Fig. 2, ANOVA, Tukey’s test, P < 0.001).

To confirm that the compounds detected by SIFT-MS were behaviorally active, we exposed living bed bugs to a synthetic mixture of hexenal and octenal in a 70:30 ratio in acetone at a concentration of 8.46 mg/mL on a paper point. The Harlan and McCall female bed bugs exposed to the synthetic AP blend responded similarly to what was observed in the living flame heated bed bug experiments (Fisher’s exact test: P < 0.05: Table 2A). Bed bugs in control experiments did not react to solvent (acetone) treated papers.

**Table 2:** Percentages of replicates where bed bugs (recipients) did or did not respond by moving in response to AP in behavioral assays, during the 5 min observation period.

	Yes, responded (%)	No, did not respond (%)	N	DF	Chi-square	P-value
(A) Treatment, female bed bugs, synthetic AP blend						
Control Harlan females, 0 mg/ml	0	100	40	1	22.9	< 0.0001
Harlan females, 8.5 mg/ml	75	25				
Control McCall females, 0 mg/ml	0	100	36	1	11.9	< 0.0001
McCall females, 8.5 mg/ml	77	22				
(B) Treatment, heated living and dead bugs, 50 mm						
Control, dead McCall female hotplate heated	14.3	85.7	41	1	6.03	0.014
Treatment, living McCall female hotplate heated	50	50				
Control, dead McCall female flame heated	19	81	40	1	8.09	0.005
Treatment, living McCall female flame heated	63	37				

Control treatments are compared to living bed bugs using logistic regression (P < 0.05). Part A of the table shows the responses of female bed bugs exposed to 8.5 mg/mL synthetic alarm pheromone solution or acetone (control) applied to paper points in still air glass funnel (50 mm) bioassays. Part B shows responses of 1:1 ratio emitter: recipient in 50 mm glass funnels when exposed to living or dead (control) bed bugs were either heated with a flame or hotplate. In all experiments for Part B, the recipients were the same sex and from the same population as the emitter.

Lastly, tests were also performed in 50 mm Petri dish-glass funnel arenas with 1:1 emitter: recipient ratio to compare the behavioral responses of bed bugs to AP released in response to different high temperature heat sources such as exposure to flame vs. high temperature hotplate (~ 300°C). The number of bed bugs responding between the two methods of heating did not differ (Logistic regression, P > 0.05; Table 2B). These results indicate flame-heating of living emitters or dead controls was not a sole contributing factor in recipient bed bugs response since hot plate heating also yielded similar results.

## Discussion

We found that bed bugs emitted AP in response to heat exposure beginning at sublethal temperatures (42 °C) and also as insects experienced lethal (44–45 °C) temperatures (Figs. 2, 3). In separate experiments, stationary bed bugs responded to AP signals emitted by heat exposed conspecifics by frantically moving (Table 1A–C). These findings suggest that at a certain threshold, bed bugs perceive heat as an indication of stressful and hazardous conditions and respond by releasing AP to alert nearby bed bugs of the lethal implications of high temperature exposure to nearby conspecifics. Alternatively, when mortality occurs in bed bugs, they may emit AP as a “death signal” to communicate a threat to nearby bugs. However, our finding that bed bugs begin emitting AP at sublethal temperatures, and that most of these individuals survive from sublethal exposure, implies that their response may not be a death indicator but a defense mechanism that acts as a warning signal that a life-threatening situation has been encountered.

### Emission of alarm pheromones in response to lethal and sub-lethal heat exposure

Bed bugs can be difficult to eliminate due to insecticide resistance, their cryptic behavior, and an inability to treat certain locations in domiciles where the insects reside^45–49^, thus heat is used to control bed bugs in sensitive areas. However, heat treatments can be challenging, as the insect may survive heat treatments used for infestation elimination^42^ or flee to adjacent unheated apartments or areas^43^. As hypothesized in the introduction section, if heat causes live bed bugs to release AP, shelter seeking or escape behavior could be induced in conspecifics that detect the pheromone signal. To determine if bed bugs emit AP after heat exposure, we used real-time SIFT-MS methods to collect and measure (E)-2-hexenal and (E)-2-octenal. Pheromone measurements revealed that both live female and male bed bugs emit AP in response to sufficient heat exposure, which is likely due to both sexes responding to elevated temperature as a life threatening condition^21–23^.

Alarm pheromone emission was not uniform among bed bugs as we found that Harlan bed bugs emitted higher concentrations of AP than the McCall population, and females more than males. Similarly, Liedtke et al.^50^ reported that levels of AP extracted from *Cimex lectularius* and *Cimex hemipterus* were highly variable and suggested that the variability could be due to differences in health, size of pheromone glands, or the extent of alarm response. In our current study, size could have influenced AP levels between sexes and populations, as Harlan females anecdotally tended to be the largest in size. We did not quantify the size of individual bed bugs used in the experiments to test this hypothesis. It is also possible for the variability to be related to bed bugs being fed 24 h before use. In this case, individuals may have fed differentially which affected the amount AP they emitted when heat stressed. In addition, laboratory conditions may also have played a role in selecting for increased AP emission in the Harlan compared to the McCall population, the former being reared in the lab since 1973 and the later collected from apartments in 2016. The exact cause(s) for the sex and population-based differences in AP emission we observed require further study.

The concentrations of AP that were emitted by bed bugs in response to sublethal heat were similar to the concentrations emitted by lethally exposed bed bugs (SI Table 1, Overall model AP emission, McCall Female Lethal vs. Sub-lethal heat). Therefore, sublethal heating of bed bugs would result in AP emission at sufficient concentrations to cause conspecifics to flee, where it may be more likely that fleeing bed bugs can finding protection as temperatures approach lethal limits. Bed bugs are more likely to be initially exposed to sublethal temperatures during a heat treatment because of the slow rate at which the room temperature increases. We also found that the release of AP in living bed bugs but not in frozen dead bed bugs, which indicates that insects must be living prior to heat exposure to emit high concentrations of AP. This finding is similar to experiments which showed that living earthworms release AP in response to electrical shock^51^, but dead earthworms do not^52^.

### Still-air bioassays with bed bugs emitting AP after heat exposure

To assess the reaction of bed bugs exposed to AP from a conspecific that had been flame-heated to induce instant mortality, we conducted still-air bioassays. We found that the AP emitted by heat exposed bed bugs caused frantic movement in recipients from both populations and sexes at 50 mm (Table 1A,B). Sustained frantic movement was seen in all the responding bed bugs during the 5 min observation period (SI video 1). Bed bugs would change directions often, and sometimes wedge themselves underneath the funnel, indicating an ability to detect differences in concentrations of the AP and desperation to escape from that area, respectively. Similar frantic movement and directional changes were seen in bed bugs exposed to their synthetic AP blend (Table 2A). The results with synthetic AP validate that the heat induced emission of bed bug volatile compounds (hexenal and octenal) detected in the SIFT-MS experiments were likely the same compounds that elicited frantic movement in the still-air bioassays.

Bed bugs may not need or use a long-range AP to alert nearby conspecifics of a threat, likely due to their preference to reside in aggregations. In support of this we found that bed bugs were less responsive in the 100 mm funnels than in the experiments with the 50 mm funnels. Although statistical differences were seen for both experiments (Table 1A,B) with the Harlan strain, the McCall strain had a similar response to controls in 100 mm funnels. This could also be due to the McCall strain emitting lower concentrations of AP compared to the Harlan strain or they may not be effectively detecting the AP at distances more than 50 mm from the emitter (Fig. 2). The lower number of responding bed bugs in the 100 mm experiments indicates a smaller active range for bed bug AP. Levinson and Bar^30^ similarly found that bed bugs react to synthetic AP at short distances.

No difference was found between the reaction of males and females in bioassays with one recipient, likely due to both sexes having similar AP olfactory neurons^50^ and therefore respond to the same to volatile compounds^25^. Although different quantities of AP were emitted by bed bugs from the two populations (Fig. 2), the overall similar reactions of Harlan and McCall bed bugs to the AP signal of emitters and different concentrations of synthetic AP (SI Table 2) implies that there is a range of hexenal and octenal concentrations that insects will respond to after a threshold is reached.

One bed bug can cause movement in multiple recipients as indicated by the experiments with five bed bugs (Table 1B). It is possible that AP emission by one bug could cause AP emission in a receiver, thus causing other bed bugs to respond. However, we did not test for this possibility as it was not the goal or objective of this study. Females were generally less responsive than males in the 1:5 emitter to recipient experiments, which has been previously reported in aggregation pheromone bioassays^27^. Females may be less responsive to AP than males in these circumstances in order to save energy for egg laying^27^. Males were more active in these experiments, possibly due to their feeding status which would cause them to attempt to mate with conspecifics^33,34^. It was observed that males would mount the dead frozen bed bug or other living males in some control replicates in an attempt to mate.

### Implications of bed bug alarm pheromone emission to heat as a management tool

For more effective bed bug elimination, integrated pest management (IPM) is used, which combines population monitoring, use of insecticides, and non-chemical control methods, such as the use of high temperatures^47,49,53,54^. Heat can be used to eliminate bed bugs by laundering or the application of steam to furniture^38–43^. Compartmentalized heaters can be used for whole domicile heat treatments or thermal remediation, which can eliminate all bed bug life stages by circulating heated air (at a temperature of ~ 60 °C) in the home for a proper length of time^41–43^.

However, our findings that bed bugs emit AP in response to sublethal and lethal heat exposure and that conspecifics respond to heat induced AP by moving, could provide a partial explanation for why sometimes a small proportion of bed bugs survive heat treatments. Consequently, AP release by heat stressed bed bugs may reduce the efficacy of heat treatments, which should be considered while designing effective strategies to control these pests. Specifically, AP may cause bed bugs to disperse to non-treated areas and avoid lethal temperatures. Moreover, our finding that bed bugs release AP at sublethal temperatures is troubling, as this implies that insects may be induced to flee from harborages before lethal temperatures have been achieved throughout treated areas and thus bed bugs may find refuge elsewhere.

Previous research has compared the heat repellency behavior of different bed bug populations when exposed to rising temperatures in their harborages^37^. These studies found that when bed bug hiding places are heated to 40–43 °C, they can flee and randomly locate unheated areas in close proximity^37^. Therefore, it is likely that during a thermal treatment, bed bugs are stimulated to move by both heat-induced repellency^34,36,37,42,43,54^ and AP emitted by conspecifics, but how bed bugs react to both in combination is unknown. When *Apis mellifera* workers are exposed to their AP at elevated temperatures, they are more active^55^ and this could also be the case with bed bugs.

Considering that fed bed bugs are repelled by heat^34,36,37,42,43,54^ and that they emit AP at temperatures that can cause knockdown (42 °C) and mortality (~ 45 °C), actions could be taken during heat treatments to prevent the insects from escaping from heated areas and relocating to thermally protected locations or the edges of a domicile. Heavily infested furniture and other items should not be placed at the perimeter of the domicile or near heat protected areas that bed bugs could easily escape to. When the interior temperatures of domiciles reach levels that would cause bed bugs to emit AP, individuals conducting treatments should monitor for bed bugs moving to thermally protected areas. However, care must be taken to ensure that individuals working indoors during a heat treatment do not experience thermal exhaustion. If cracks and crevices or other areas that bed bugs are relocating to are found, those areas need to be treated with insecticide or sealed in advance to prevent escape.

Our behavioral experiments with bed bug pheromones indicate that synthetic AP could be utilized within an IPM program. Application of synthetic AP at low concentrations could be potentially used to (i) flush bed bugs out of resting areas where it is difficult to elevate temperatures above sublethal levels to places that experience lethal heat and (ii) monitor and test for the presence and location of living insects before and after heat treatments. Further research is necessary to determine the practicality of using bed bug AP for control purposes and how AP causes bed bugs to move during a heat treatment.

## Methods

### Insects

Two populations were used in the study, the insecticide susceptible Harlan population and a more recently collected McCall population from Florida. The Harlan population has been maintained in the laboratory since 1973 and was originally collected from the wild in Fort Dix, NJ. The McCall population was collected in 2016 and was reared in the lab for approximately 20 generations before used in the experiments conducted in the current study. The McCall population was used because it was collected from a domicile in Florida after a heat treatment and, thus, it had presumably survived thermal exposure in the field^44^. Also, the McCall population has experienced a relatively short period of rearing under laboratory conditions compared to the Harlan population and thus its sensitivity to emitting and responding to AP may more closely reflect those of insects found natural settings. Both bed bug populations were maintained at 26 ± 1 °C, 50 ± 10% RH and a 12:12 h (L:D) cycle in a temperature-controlled environmental chamber (Percival Scientific, Perry, IA). Bed bugs were fed on defibrinated rabbit blood (Hemostat Labs, Dixon, CA) heated to 37 °C using the membrane feeding method^56^ 24 h prior to experimentation. Fed bed bugs were used in all experiments because sublethal temperatures up to 38 and 43°C may stimulate unfed insects to be more prone to move in search for blood meals than fed insects^36^, confounding behavioral observations.

### Detection of volatile AP components emitted from bed bugs exposed to lethal and sublethal heat

To determine when bed bugs emit AP in response to heat exposure, headspace volatiles were continuously sampled from a single bed bug placed on a digital hotplate (ThermoFisher Scientific, Wilmington, DE) underneath an inverted 50 mm glass Pyrex funnel apparatus (Corning Life Sciences, Oneonta, NY) (Fig. 1). Volatile collection experiments were conducted from June to August in 2018, November 2019, and February 2020. The SIFT-MS (selected ion flow tube mass spectrometry) took air samples through the stem of the funnel every 5 s (Syft Technologies, Christchurch, NZ). Based on measurements taken using an air flow calibrator (Defender 510, Bios International Corporation, Butler, NJ), the volumetric air flow through the SIFT-MS was determined to be 27.91 mL/min. The SIFT-MS allowed for real-time concentration measurements of (E)-2-hexenal and (E)-2-octenal present in head space volatiles, the two compounds previously shown to be the active components of bed bug AP^22^. The reagent ions H3O +, NO + and O2 + (SIFT-MS calibrated standard gas) were generated in the ion source by a microwave discharge operating in low pressure moist air. The reagent ions formed in the microwave discharge were mass selected by the upstream quadrupole and injected into the flow tube, where they were carried along the tube in a stream of helium. The concentrations of (E)-2-hexenal and (E)-2-octenal were automatically determined using software (LabSyft, Pittsburgh, PA) on the SIFT-MS computer, which calculates the aldehyde concentrations based on the mass of differences in the calibrated standard gas (Airgas, Indianapolis, IN). Chemical analysis was performed using selected ion mode (SIM) scan for only (E)-2-hexenal and (E)-2-octenal. Additional details concerning the general methodology of SIFT-MS can be found in Spanel and Smith^57^.

Volatiles were collected from bed bugs for 30 total mins in two phases for the lethal heat experiments. For the first 15 min constituting phase one, one adult bed bug (male or female, from either the Harlan or McCall population) was placed under the 50 mm funnel on a digitally controlled hotplate at 24 °C (i.e., near ambient or room temperature). Two thermocouples (Digi-sense, Vernon Hills, IL) attached to the left and right side of the hot plate recorded temperature (Fig. 1). Phase one provided time for a test bed bug to acclimate to the experimental apparatus and reduce stress induced from being transferred to the hot plate. For the next 15 min in phase two, the temperature was ramped up from 24 to 55 °C at a rate of 1.86 °C/min. This rate of heating is faster than a thermal treatment for bed bug infestations. However, for practical purposes a faster rate of heating was required to make observations in a reasonable timeframe. Ramp-up heat was used because it is similar to how heat is used to control bed bugs in domiciles^41^. Heated controls (i.e., [i] previously frozen or dead McCall and Harlan males and females exposed to heat and [ii] empty funnel) were treated identical to the living bed bugs as mentioned above for recording their AP values. Unheated control treatments (i.e., [i] non-heated McCall and Harlan males and females, and [ii] non-heated empty blank funnel) were treated as above for recording their AP values but were not heated and exposed to room temperatures (24 °C) for the 30-min testing period. The temperature was recorded every 50 s while the AP concentration was recorded every 5 s by SIFT-MS. Hot plate temperatures were also recorded when bed bugs began to emit AP and returned to baseline levels. Each treatment was replicated 6 times.

To determine if bed bugs emitted AP in response to sublethal heat exposure, similar methods as described above were used. One difference was that when the temperature of the hotplate reached 42 °C, this temperature was maintained for about eight minutes. This exposure time allowed us to observe both when AP emission began and when the AP concentration returned to baseline levels for the sublethal heat experiments. The total duration of the sublethal heat experiments in phase two of test starting from the initiation of heat ramping was 15–16 min. Control treatments for the sublethal heat experiments were (i) living unheated McCall females, (ii) heated dead McCall females, and (iii) empty heated blank funnels. Temperature of AP emission was also recorded. Each treatment was replicated 6 times.

Following the lethal and sublethal heat experiments, the bed bug was removed from the hotplate, placed in a 35 × 10 mm plastic Petri dish (Greiner Bio-One, Monroe, NC) in an environmental rearing chamber. Mortality was scored 24 h later by prodding the bed bug and if it did not move, it was scored as dead.

### Determining the response of bed bugs to AP emitted by heat exposed individuals in 50 mm glass funnels

To determine if recipient bed bugs reacted to AP emitted by a heat-exposed bed bug (emitter), an inverted funnel design like the headspace volatile pheromone collection apparatus was used (SI Fig. 1). For these experiments, the 50 mm funnel (28 mL funnel volume) was placed on a glass Petri dish lid (60 mm; Grainger, Indianapolis, IN) lined with a Whatman No 1. filter paper (GE Healthcare, Chicago, IL) (SI Fig. 1). The behavioral response experiments were conducted using two different ratios of emitters to recipients, 1:1 or 1:5. In all experiments, the emitter was of the same sex and from the same population as recipients.

To set up the 1:1 emitter to recipient ratio experiments, the recipient bed bug (one blood-fed bed bug, male or female from the Harlan or McCall population) was placed in a Petri dish lid and an inverted 50 mm funnel was placed over the dish. The recipient bed bug was allowed to acclimate in the setup for 24 h in lighted conditions because heat treatments are done in lighted conditions. After feeding, individual emitter bed bugs were placed into 35 × 10 mm plastic Petri dishes with Whatman No. 1 filter paper for 24 h prior to use. Separating emitter bed bugs into individual containers ensured that they did not interact with other insects which could have led to AP emission (e.g., male emission of AP in response to being mounted by another male in an attempt to mate). Control emitter bed bugs were placed in a freezer for 24 h prior to use after a bloodmeal. Before experiments, control emitters were removed and allowed return to room temperature which required 15 min. To heat expose a living emitter, an individual bed bug was grasped with feather-tip forceps by the head region and quickly passed through a flame three times (~ 600–1400 °C) and then dropped through the funnel stem, into the bioassay arena (SI Fig. 1). Briefly passing the living emitter bed bug through flame caused instant mortality and AP release, as determined by SIFT-MS in additional confirmatory experiments (see below). For the next 5 min, behavioral observations were made on the recipient bed bug to determine if it left the resting state, defined as a non-moving position (SI video 1). For the control treatments, the previously frozen dead control bed bug was not heated and was dropped into the funnel through the stem. Statistical testing was performed by logistic regression analysis on the number of replicates where recipient bed bugs moved or did not move during the experimental period. Sustained rapid movement was observed in the recipient bugs that reacted to the AP from emitters and scored as “Yes, responded” to AP. If they did not respond, they were scored as “No, did not respond”. Each treatment was replicated 20 times.

In separate experiments, it was verified that temperature of the flame heated bed bug started at ~ 76.6 °C had returned to near room temperature (26.6 °C) 15 s after exposure, thus the recipients were not exposed to heat from emitters. Temperatures of emitters was measured by placing a thermocouple (Fisher-Scientific, Hampton, NJ) directly on the heated emitter bed bug.

Procedures used for conducting the 1:5 emitter to recipient ratio experiments were identical to those mentioned above for the 1:1 ratio bioassay. In the control treatment groups, one dead frozen bed bug that had returned to room temperature was dropped into a still air bioassay with five bed bug receivers. Treated and control groups were replicated 20 times. The number of bed bugs that responded was scored. If a receiver disturbed another receiver and caused them to move, it was not scored as responding to the AP.

### Determining the response of recipient bed bugs to AP emitted by heat exposed bed bugs in 100 mm funnels

Behavioral assays for AP response were performed in an inverted 100 mm glass Pyrex funnel, in addition to a 50 mm funnel experiments described above. Aside from the larger arena size and the 100 mm assays (235 mL funnel volume) only being conducted for only a 1:1 ratio of emitter to recipient in the arena, all other aspects of this test were identical to those described for 50 mm funnel experiments. Each treatment type was replicated 18–20 times and binary yes versus no behavioral response data was generated.

### Additional validation experiments

To confirm that flame heated beg bugs emit AP comparable to hot plate heated insects we used a combination of the above mentioned SIFT-MS and still air bioassay methods. Briefly, living adult female bed bugs from the Harlan and McCall populations were fed 24 h in advance of use. Approximately half of these blood-fed bed bugs were frozen in a freezer. The next day, both living and frozen dead bed bugs were flame-heated as described above and placed under the glass funnel setup (Fig. 1) for 5 min to quantify AP levels emitted from the insects (SI Fig. 2). The SIFT-MS was used in selected ion mode (SIM) scan for (E)-2-hexenal and (E)-2-octenal.

Behavioral experiments were also conducted in 50 mm funnels using synthetic AP blend chemicals to determine if beg bugs responded similarly to the synthetic blend as AP emitted by heat treated insects. A mixture of (E)-2-hexenal (98% pure) and (E)-2-octenal (94% pure) was created in a 70:30 volume ratio (Sigma-Aldrich, St. Louis, MO). The stock solution was then serially diluted in acetone to create an 8.46 mg/mL (0.85%) solution (equal to 0.6 µg of AP blend/mL of air in 50 mm). One fed Harlan or McCall female bed bug was placed in a 50 mm glass Pyrex funnel as mentioned above. Only females were used for synthetic AP experiments because both sexes and populations had a similar behavioral response to AP emitted by conspecifics. Harlan or McCall female bed bugs were allowed to acclimate in the setup for 24 h. Next, a 1 cm by 0.3 cm strip of Whatman No 1. Filter paper was treated with 2 µL of the 8.5 mg/mL AP solution or acetone (control). Once the acetone had evaporated (10 s), the strip of paper was dropped through the stem of the funnel. For the next 5 min, behavioral observations were made for both the treated and control groups. The synthetic blend experiment was replicated 16 times each for both the Harlan and McCall populations in tandem with acetone control groups (Table 2A).

Comparative behavioral experiments were conducted using emitter bed bugs exposed to heat either on a hot plate or on flame. For these experiments, both living and dead emitter female bed bugs from the McCall populations were heated. Behavioral assay procedures for the 1:1 emitter to recipient ratio tests were identical to those described above. One group of emitters (living or previously frozen dead bed bugs) were heated on a hotplate set to ~ 300 °C for 3 s and then dropped into separate 50 mm glass Pyrex funnel setups with one recipient. The other group of emitters (living or previously frozen dead bed bugs) was heat-exposed by the flame heating methods mentioned above and then dropped into separate 50 mm glass funnel setups with one recipient. Binary response data was collected. Hotplate experiments were replicated 21 times and the flame heated experiments were replicated 30 times (Table 2B).

### Data analysis

To compare the AP emission data from the different treatment groups, which were treated as a single category to reduce explanatory variable (living Harlan and McCall bed bugs and different controls), the concentrations of hexenal and octenal (mg/mL of air) measured by SIFT-MS were first summed at each observation point. This was to simplify the analysis and because bed bugs would experience both compounds simultaneously. Only the AP data for timepoints shortly before, during, and after the AP spike, usually for a period of 600 s, were used for analysis. Control treatments (unheated empty funnel, heated empty funnel, heated dead bed bugs, unheated live bed bugs) used the time point data corresponding to the heated living bed bug treatments for statistical comparisons. For presentation, we display the entire dataset of hexenal and octenal in response to different temperatures to show that the compounds were not detected outside of the timepoints that were statistically compared. An ANOVA and post hoc Tukey’s tests were used to compare the summed AP concentrations for each treatment group in JMP Pro 16.

To analyze the response of recipient bed bugs to AP released by heat-exposed emitter bed bugs in the 50 mm and 100 mm glass funnels, pair-wise logistic regression was conducted on the total number of insects that responded or did not respond, for each treatment type. Controls of each group (sex and population) were compared to its corresponding heated treatment type. The same analysis was used to compare the response of conspecifics to living and dead bed bugs that were heated with either a flame or hotplate and synthetic AP.

### Supplementary Information

Supplementary Information.

Supplementary Video 1.

## Data Availability

Data will be made available upon request, please contact A.R.A.
