# Supplementary material for: Lethal and sublethal heat-exposure of bed bugs (Cimex lectularius L.) causes alarm pheromone emission and elicits a movement response in nearby recipients
Source: Sci Rep. 2024 Apr 12;14:8555. doi: 10.1038/s41598-024-57925-y (PMC11014846; doi:10.1038/s41598-024-57925-y)
Supplement: Supplementary file 1 — Supplementary Information. [file 41598_2024_57925_MOESM1_ESM.pdf]

SI Table 1. Statistical details for various ANOVA analyses. DF stands for degrees of freedom.

Data used for ANOVA analysis	DF	Sum of squares	F-Ratio	P-Value
Overall model AP emission by bed bugs lethal heat, aldehydes over time	23	0.01	9.96	<0.0001
AP emission by bed bugs, time	1	0.001	31.7	<0.0001
AP emission by bed bugs, treatment group	11	0.005	8.8	<0.0001
AP emission by bed bugs, treatment group*time	11	0.005	8.9	<0.0001
AP emission by bed bugs, treatment group*temperature	1	0.00004	2.7	0.09
Overall model AP emission by bed bugs sublethal heat, aldehydes over time	7	0.0001	2.09	0.045
AP emission by bed bugs sublethal heat, time	1	0.000005	0.5	0.46
AP emission by bed bugs sublethal heat, treatment group	3	0.0001	4.2	0.0062
AP emission by bed bugs sublethal heat, treatment group*time	3	0.00002	0.5	0.7
Overall model AP emission, live vs. dead flame heated	487	0.006	347.0	<0.0001
AP emission, live vs. dead flame heated*group	7	0.004	155.1	<0.0001
AP emission, live vs. dead flame heated*time	60	0.005	2.4	<0.0001
AP emission, live vs. dead flame heated group *time	420	0.002	1.3	<0.0001
Overall model AP emission, McCall Female Lethal vs. Sub-lethal heat	131	0.0004	2.2	0.10
AP emission, McCall Female Lethal vs. Sub-lethal heat, group	1	0.00002	0.33	0.56
AP emission, McCall Female Lethal vs. Sub-lethal heat, time	1	0.0002	3.1	0.08
AP emission, McCall Female Lethal vs. Sub-lethal heat, group*time	1	0.00003	0.5	0.47

SI Table 2. Percentage of replicates with bed bugs that responded to the synthetic AP blend at different concentrations. The AP concentration produced in the 50- and 100-mm funnels is also listed, but synthetic AP experiments were only conducted in the 50mm funnels[58]. Experiments were conducted as described in the synthetic AP methods section.

Concentration of AP mixture (mg/mL)	Concentration of AP mixture (µg/mL of air) in 50 mm funnels	Concentration of AP mixture (µg/mL of air) in 100 mm funnels	Percentage Responding
0	-	-	5%
0.085	0.0061	0.0007	50%
0.85	0.061	0.007	75%
4.2	0.3	0.036	69%
6.6	0.47	0.056	46%
8.5	0.61	0.07	75%
13.2	0.95	0.11	77%
26.4	1.89	0.22	83%
52.8	3.78	0.45	86%
105.7	7.55	0.9	57%

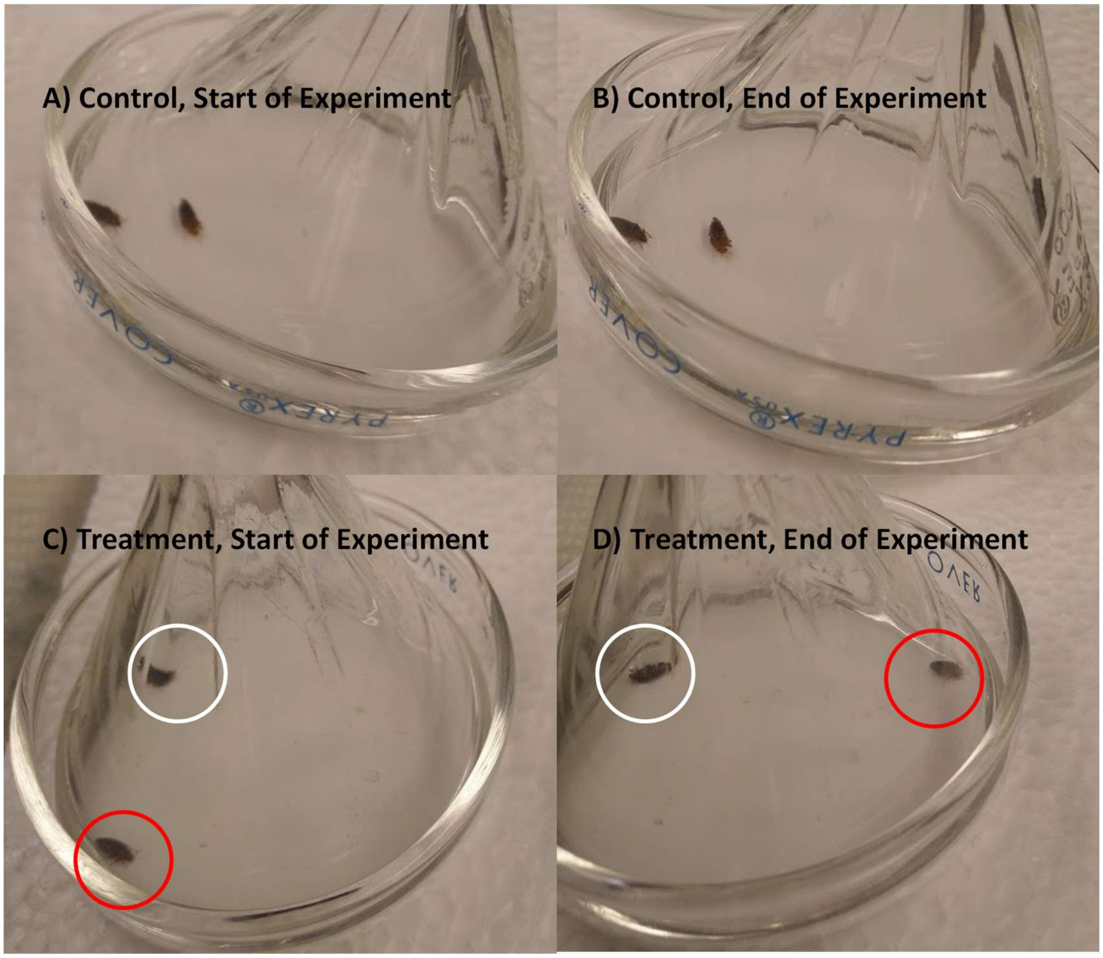

SI Figure 1. The 50 mm glass Pyrex® funnel apparatus for evaluating behavioral responses of bed bugs to alarm pheromone (AP) released by lethally heat exposed emitter bed bugs. (A, B) The control funnels with a previously frozen dead bed bug inside, there was no change in location in the recipient bed bug. (C, D) Heated emitter bed bug indicated by white circle in the funnels on the left and the recipient bed bug in red circle, which responded with frantic movement during the 5-minute observation period.

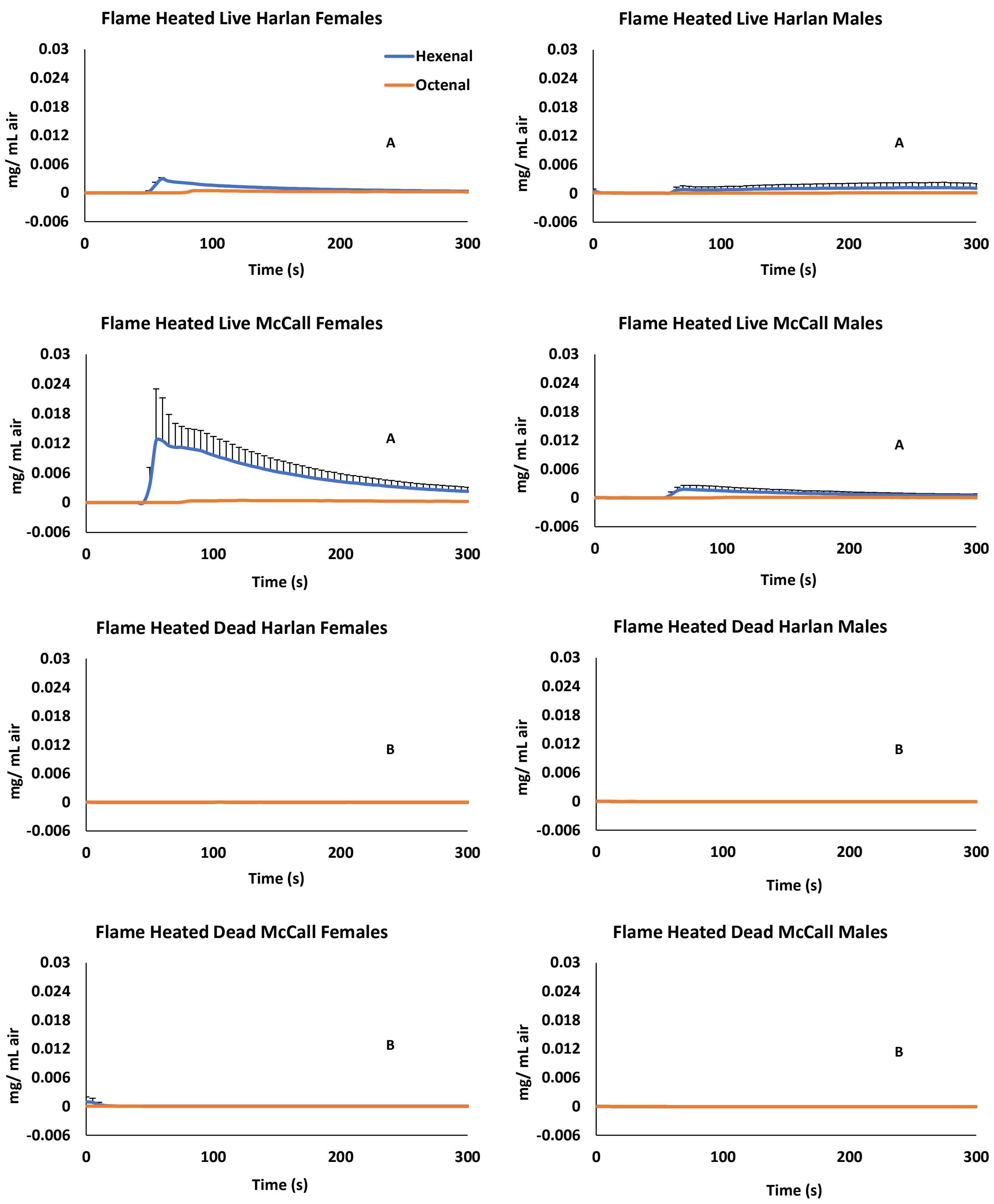

SI Figure 2. Averages of alarm pheromone (AP) emission profiles of live and previously frozen deceased adult female bed bugs from the Harlan and McCall populations in response to being flame heated as detected by the soft ion flow tube mass spectrometer SIFT-MS. The blue lines represent the average detected amount of hexenal, whereas the orange lines represent the average concentration of octenal. Live flame heated bed bugs were found to have significantly different quantities of hexenal and octenal (ANOVA, t-test, $P<0.05$) compared to the flame heated dead bed bug groups. Error bars represent standard deviation values. Each treatment was replicated three times.

VIDEO0006.mp4

SI Video 1. The video depicts the “excited as well as repelled by” or frantic response of the receivers when exposed to AP from live bed bugs that were killed using heat. When this behavior was observed, the bed bug was scored as responding to the alarm pheromone.
